# Wheat Ears Counting in Field Conditions Based on Multi-Feature Optimization and TWSVM

**DOI:** 10.3389/fpls.2018.01024

**Published:** 2018-07-13

**Authors:** Chengquan Zhou, Dong Liang, Xiaodong Yang, Hao Yang, Jibo Yue, Guijun Yang

**Affiliations:** ^1^School of Electronics and Information Engineering, Anhui University, Hefei, China; ^2^Key Laboratory of Quantitative Remote Sensing in Agriculture of Ministry of Agriculture P. R. China, Beijing Research Center for Information Technology in Agriculture, Beijing, China; ^3^National Engineering Research Center for Information Technology in Agriculture, Beijing, China; ^4^International Institute for Earth System Science, Nanjing University, Nanjing, China

**Keywords:** superpixel theory, multi-feature optimization, support-vector-machine segmentation, wheat ear counting, yield estimation

## Abstract

The number of wheat ears in the field is very important data for predicting crop growth and estimating crop yield and as such is receiving ever-increasing research attention. To obtain such data, we propose a novel algorithm that uses computer vision to accurately recognize wheat ears in a digital image. First, red-green-blue images acquired by a manned ground vehicle are selected based on light intensity to ensure that this method is robust with respect to light intensity. Next, the selected images are cut to ensure that the target can be identified in the remaining parts. The simple linear iterative clustering method, which is based on superpixel theory, is then used to generate a patch from the selected images. After manually labeling each patch, they are divided into two categories: wheat ears and background. The color feature “Color Coherence Vectors,” the texture feature “Gray Level Co-Occurrence Matrix,” and a special image feature “Edge Histogram Descriptor” are then exacted from these patches to generate a high-dimensional matrix called the “feature matrix.” Because each feature plays a different role in the classification process, a feature-weighting fusion based on kernel principal component analysis is used to redistribute the feature weights. Finally, a twin-support-vector-machine segmentation (TWSVM-Seg) model is trained to understand the differences between the two types of patches through the features, and the TWSVM-Seg model finally achieves the correct classification of each pixel from the testing sample and outputs the results in the form of binary image. This process thus segments the image. Next, we use a statistical function in Matlab to get the exact a precise number of ears. To verify these statistical numerical results, we compare them with field measurements of the wheat plots. The result of applying the proposed algorithm to ground-shooting image data sets correlates strongly (with a precision of 0.79–0.82) with the data obtained by manual counting. An average running time of 0.1 s is required to successfully extract the correct number of ears from the background, which shows that the proposed algorithm is computationally efficient. These results indicate that the proposed method provides accurate phenotypic data on wheat seedlings.

## Introduction

Wheat is an important primary food for a large proportion of the world's population, so methods to estimate its yield have received significant research attention (Bognár et al., 2017). The number of ears per unit area, the number of grains per ear, and 1000 grain weight are known as the three elements of wheat yield (Plovdiv, 2013). Of these, the number of ears per unit area is mainly obtained in the field. The wheat ear is an important agronomic component (Jin et al., 2017) not only is closely associated with yield but also plays an important role in disease detection, nutrition examination, and growth-period determination. Thus, an accurate determination of the number of ears is vital for estimating wheat yield and is a key step in field phenotyping (Zhang et al., 2007). At present, two main statistical methods exist to obtain the number of ears per unit area: manual field investigation and image-based crop recognition (Nerson, 1980). Manual field investigation, which is the traditional method, is inefficient and costly, resulting in more and more interest in image-based crop recognition. However, because of the complexity of the field environment (e.g., illumination intensity, soil reflectance, and weeds, which alters the colors, textures, and shapes in wheat-ear images), accurate wheat ear segmentation and recognition remains a significant challenge (Mussavi and M. Sc. of Agronomy Ramin Agricultural and Natural Resources, 2011). In the field of image segmentation, a number of meaningful research results have emerged in recent years. These methods mostly focus on two approaches, the first of which is based solely on color information (Naemura et al., 2000). For example, Chen et al. proposed a threshold-selection algorithm for image segmentation based on the Otsu rule (Chen et al., 2012). Subsequently, Khokher et al. introduced an efficient method for color-image segmentation that uses adaptive mean shift and normalized cuts (Khokher et al., 2013). Moreover, Liao et al. used an edge-region active contour model for simultaneous magnetic resonance image segmentation and denoising (Liao et al., 2017). Additionally, the color information for wheat changes over the reproductive stage. Thus, different methods usually apply to different stages of reproduction. Therefore, in addition to the disadvantages described above, an excessive dependence on color information will lead to incomplete extraction.

The second approach involves machine learning. For example, Kandaswamy et al. used the meta-segmentation evaluation technique to deal with the problem of image segmentation (Kandaswamy et al., 2013). Linares et al. introduced an image-segmentation algorithm based on the machine learning of features (Linares et al., 2017). Soh et al. proposed a method based on a linear classifier that reveals a new method of segmentation (Soh and Tsatsoulis, 1999). In addition, Lizarazo et al. used a support vector machine (SVM) classifier to segment remote-sensing data (Lizarazo, 2008). Because of its high accuracy and robustness, target segmentation based on classifiers was widely used for target recognition in the field of complex environments (Lizarazo and Elsner, 2009). This method mainly includes two key steps: (i) extraction and combination of image features and (ii) selection of classifiers to be trained.

The first step above forms the basis of image recognition (Song et al., 2016). Choosing the appropriate features directly impacts the final segmentation and recognition accuracy (Ding et al., 2017). Hu et al. proposed an image-feature-extraction method based on shape characteristics (Hu et al., 2016), and Yang et al. introduced multi-structure feature fusion for face recognition based on multi-resolution exaction (Yang et al., 2011). Datta et al. applied kernel principal component analysis (KPCA) to classify object-based vegetation species to fuse color and texture features, which has good results (Datta et al., 2017). To summarize, compared with the single-feature method, using a variety of features to express the red-green-blue (RGB) images can be more comprehensive and effective for improving the descriptive ability.

Next, another key step of the classifier-based segmentation method is to use a general classifier to classify the features. The representative image classifier to be trained mainly includes a rough set, a Bayesian, and a SVM. Banerjee et al. used rough set theory to solve the problem of multispectral image classification (Banerjee and Maji, 2015). Zhang et al. proposed a method for multiple categories based on Bayesian decisions (Zhang et al., 2014). Finally, Park et al. introduced an automatic image-segmentation method that uses principal pixel analysis and SVM (Park et al., 2016). Upon comparing with the other two classifiers, the SVM proves simpler in structure and offers global optimality and good generalization, so it has been widely used in the fields of image recognition and classification. However, the speed with which SVM learns a model is a major challenge for multi-class classification problems.

To overcome these problems, the present study proposes a segmentation algorithm based on multi-feature optimization and twin-support-vector-machine (TWSVM) (Jayadeva et al., 2007). First, the algorithm extracts the color feature, texture feature, and edge histogram descriptor of wheat-ear images. Second, we use the KPCA to obtain the corresponding weights for each feature to rationally construct the feature space. The feature space is composed of multiple features to more comprehensively describe the target images, through which the advantages of each feature for classifying the different classes are manifested. Finally, the training of the TWSVM model is completed and better performance is obtained.

The remainder of this paper is organized as follows: The next section describes in detail both the study area and image preprocessing. Section Methods describes the methodology. Section Results describes the experimental results and demonstrates the robustness of the method. Finally, we finish the paper with concluding remarks and possible directions for future work.

## Study area and data preprocessing

### Study area

**Figure 1:**
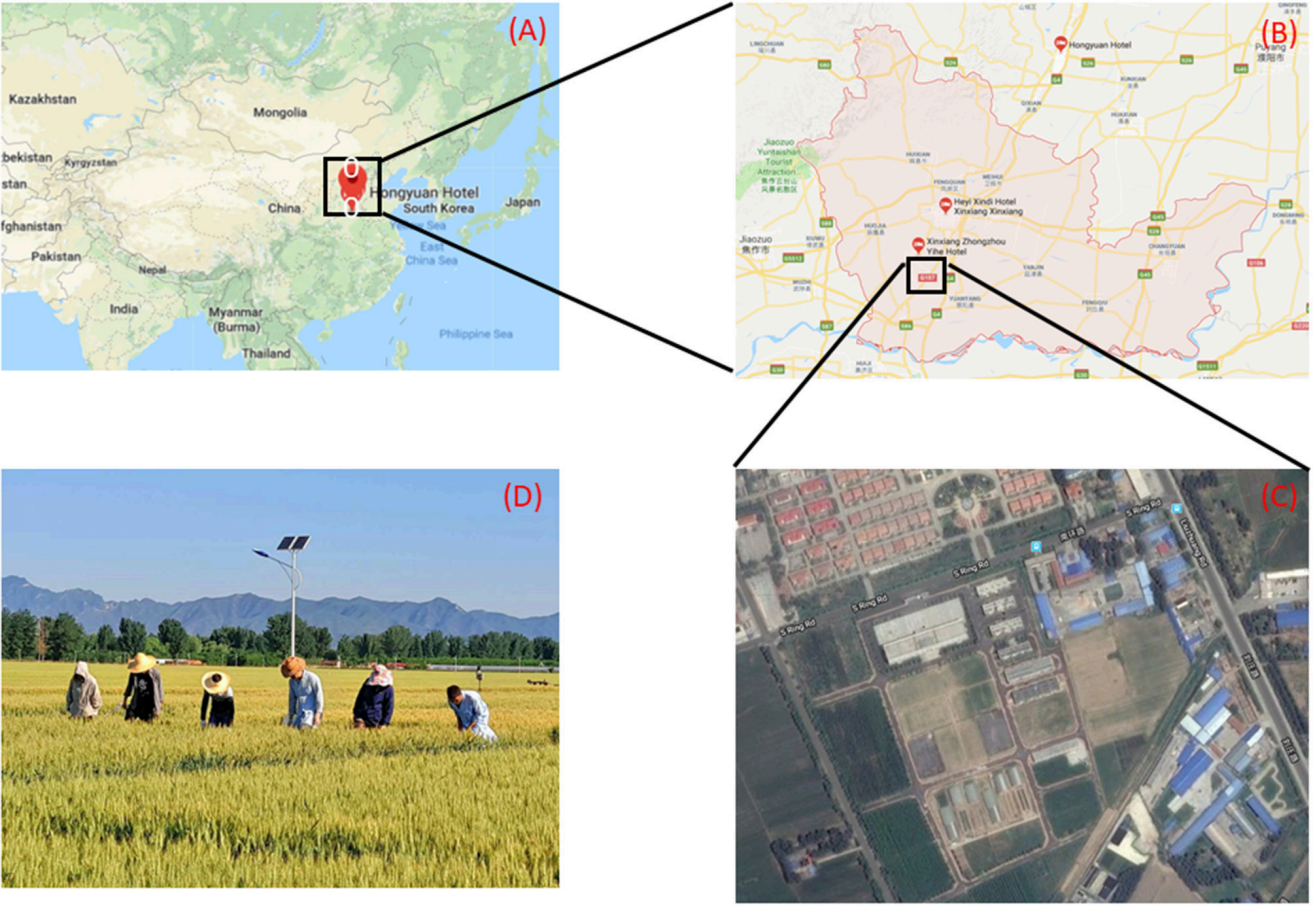
**(A)** Location of basement in China. **(B)** Location of basement in Xinxiang City. **(C)** Satellite map of experimental area. **(D)** Working state in the field.

The field planted with wheat was located in the Xinxiang comprehensive experimental base of the Chinese Academy of Agricultural Sciences. (Xinxiang, China, 35°9′32″ latitude North, 113°48′28″ longitude East). The sowing date was October 16, 2015. The experiment was conducted from 10 a.m. to 2 p.m. on June 9, 2016. For this paper, we collected data in overcast weather conditions, which resulted in totally scattered skylight with no direct illumination. These conditions eliminate shadows. While obtaining image data in the field, we made manual ground measurements of the corresponding plot to obtain manual-recognition data at the same time. The manual investigation area is 4 m^2^ in each plot and the total area covered is 1200 m^2^ with 300 plots. (Figure 1).

### Data acquisition and preprocessing

#### Image acquisition

**Figure 2:**
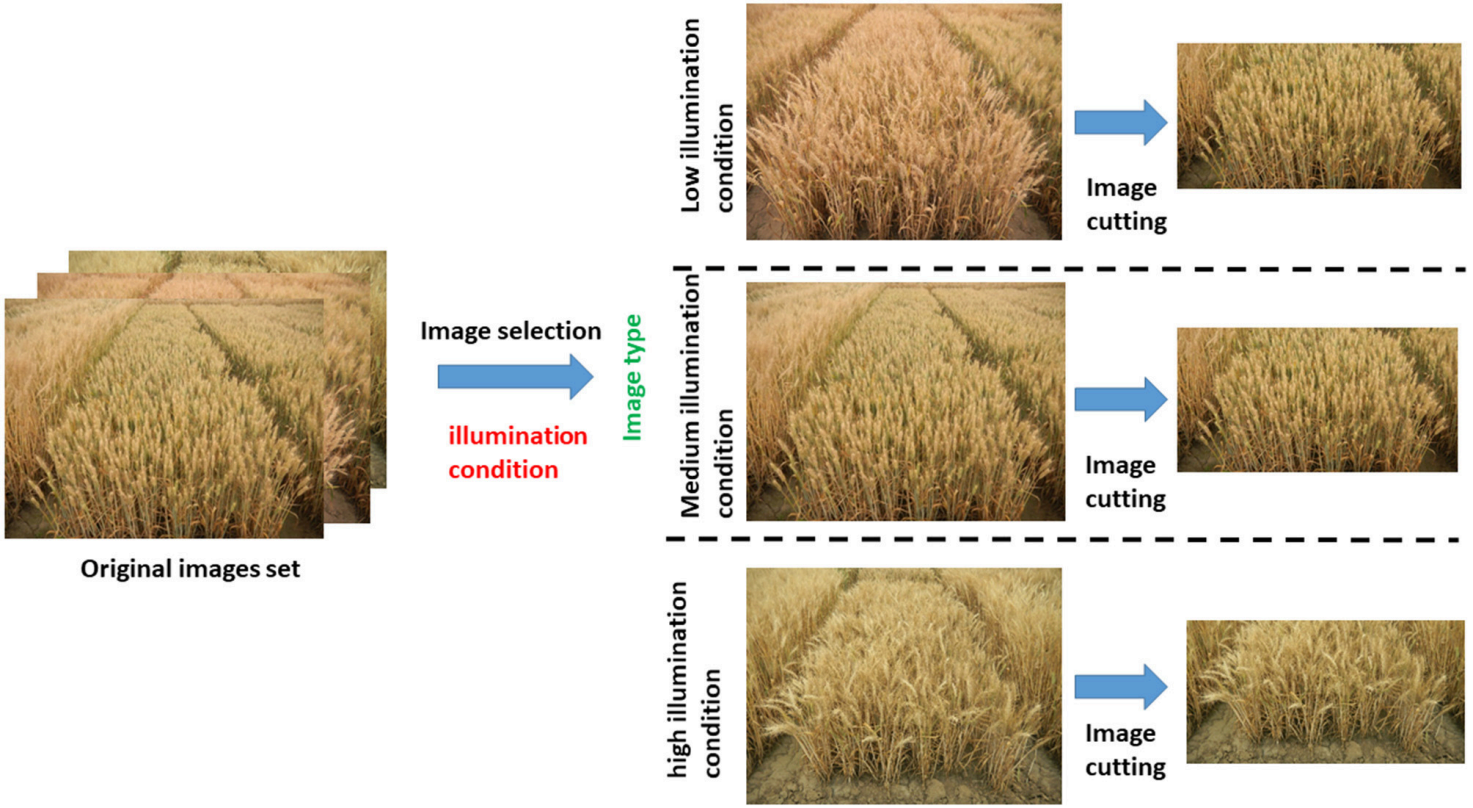
Preprocessing strategy for original image.

For each observation of an individual wheat ear, we systematically varied the illumination factors. Figure 2 shows an example of an image collected during a single observation. We imaged wheat ears from the side at 45° above the horizontal because color and texture are typically substantial from this perspective. The camera aperture was f/3.9 with an exposure time of 1/90 s. The focal length of the camera was 50 mm.

#### Image preprocessing

(A) Selection of dataset samples

In conditions of varying illumination intensity from morning to afternoon, 1000 images were obtained with the same shooting mode (2 m imaging distance, 1.5 m imaging height, imaging angle at 45° above horizontal) and the same camera parameters as mentioned above. As a result of the limitation of the number of sample images, 700 images were used as the training sample and the rest 300 images were used as the testing sample. This procedure gave us images under differing light intensities. We next divided these images into the following three categories by visual analysis: (a) high light intensity, (b) medium light intensity, and (c) low light intensity. All the images were selected from each category as the source of training set. This processing guarantees the robustness of the light intensity of the training results.

(B) Image cutting

The images used in this work were all obtained from oblique photography. As a result of the perspective, the wheat ears far from the shooting position are not well rendered in the images. The limitations imposed by camera resolution and the position of the camera focus make this part of the image low quality, so we cut the image to remove these parts and ensure uniform data quality. After this cutting process, the image size was reduced to 3500 × 1800 (Figure 2).

(c) Counting results validation

The performance of the image processing system to automatically counting the ears appearing in an image was tested in the images. In order to validate the algorithm, the *machine counting result* was compared with the manual image-based ear counting on the same image. *Machine counting result* depicts the binary image where the connected pixels in white color are considered as a wheat ear automatically detected by the image processing system; each of these regions are added and the final result is referred to as the algorithm counting. Besides, the number of ears in a subset of images has been counted manually and is referred to as the *manual counting result*. To ensure the precision of the manual statistics, two people repeated the counting operation according to the field range of the cut images. Moreover, in order to judge the accuracy of the segmentation, the wheat ear area is manual labeled as red small block. Then the labeled images were used as the mask images to compare with the machine segmentation and recognition results in order to ensure the accuracy of the method.

## Methods

**Figure 3:**
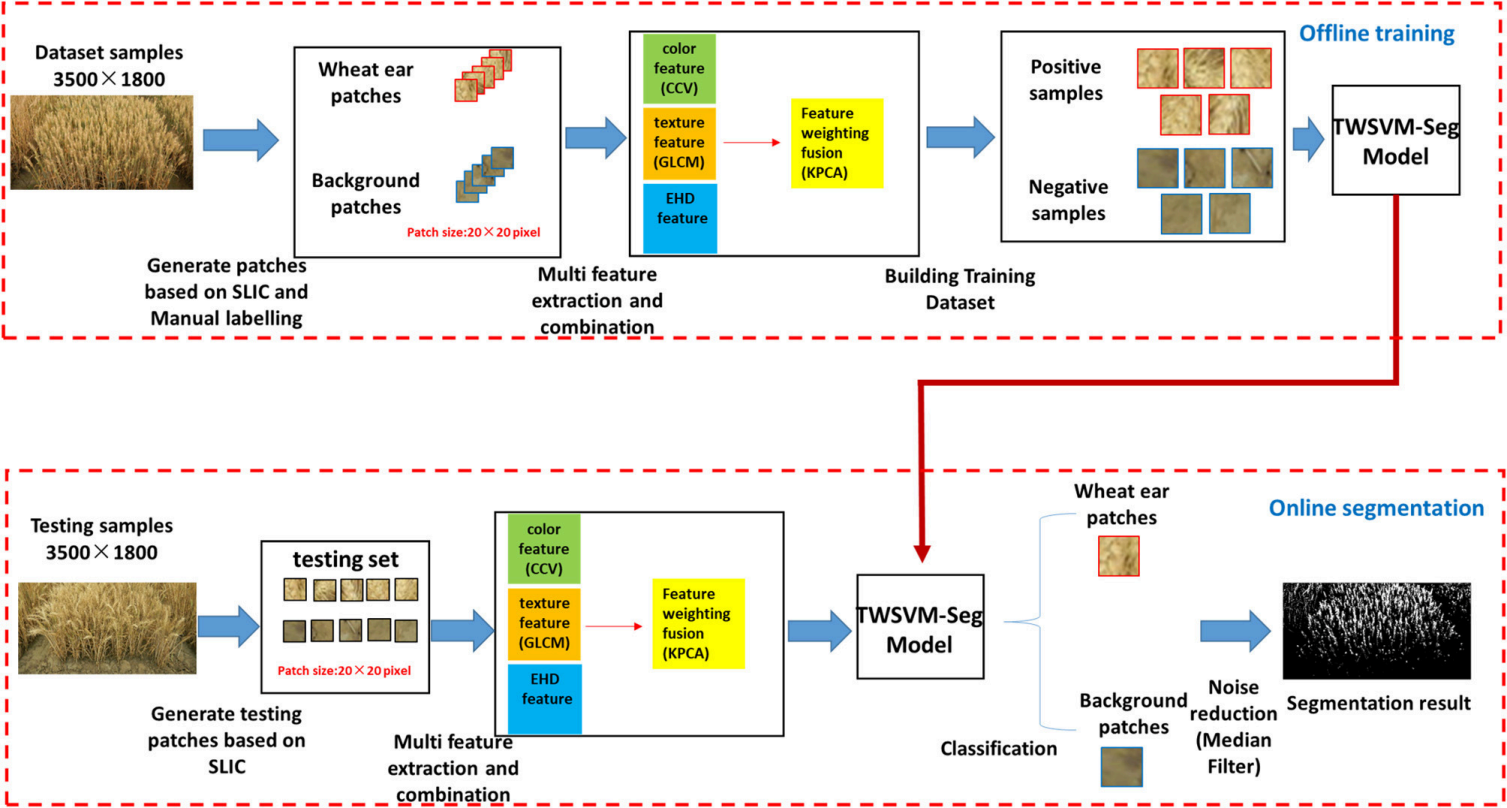
Schematic representation of method.

After image acquisition, the main flow diagram of the proposed method includes off-line training of on-line segmentation, as shown in Figure 3. This research framework consists of five consecutive steps: (i) generating patches, (ii) establishing training and test sample sets, (iii) optical combination of multi-features space, (iv) training a classifier, and (v) noise reduction. Below, we discuss each step in detail and refer in particular to the variables, image types, and preprocessing strategies that we studied in our experiments.

The specific algorithm (workflow) is as follows:

Step 1: Select *N* images as training samples and extract patches of a certain size (20 × 20) from these samples;
Step 2: Extract the color feature, texture feature, and edge histogram descriptor feature from the samples;
Step 3: Use KPCA to extract the principal component features and calculate the weight for each feature in each class of samples;
Step 4: Train the TWSVM classification model with the weighted features updated in Step 3;
Step 5: Perform a weighting to the feature in the test sample with feature weights in each class, use the TWSVM-Seg model obtained in Step 4 to classify, and determine the image segmentation (Figure 3).

### Generation of patches based on simple linear iterative clustering method and training set and validation set building

Pixel-level segmentation approaches have achieved a moderate degree of success. At the same time, ignoring the neighborhood information seriously impacts the edge-preservation of the segmentation algorithm. Thus, processing the image patches with similar characteristics instead of single pixels contributed to overcoming the influence of noise, accelerating the processing speed, and improving edge-preservation. Moreover, the TWSVM requires uniform-sized images as input. To achieve this goal, simple linear iterative clustering (SLIC) was applied to extract superpixel image patches (Achanta et al., 2012). The computing speed is faster than the other superpixel generation algorithm, and the algorithm offers superior edge preservation. SLIC is an adaptation of k-means for superpixel generation, with two important distinctions:

1. The number of distance calculations in the optimization is dramatically reduced by limiting the search space to a region proportional to the superpixel size. This reduces the complexity to be linear in the number *N* of pixels—and independent of the number *k* of superpixels.
2. A weighted distance measure combines color and spatial proximity while simultaneously controlling the size and compactness of the superpixels.

However, these SLIC superpixel regions are irregularly shaped, so they cannot be used directly as TWSVM input. Therefore, a small window called a patch (20 × 20 pixels) and centered on the weighted center of the current SLIC superpixel region is given to the TWSVM. Note that the code to implement the SLIC operation is based on open source code provided available at https://ivrl.epfl.ch/research/superpixels. After the SLIC generates the irregular superpixel regions as discussed above, the center of the patch is determined by the weighted center of the region. Then a regular patch is built according to the position of the center point. The percentages in each patch represent the ratio between the wheat ear area to the corresponding areas of the SLIC superpixel region. The sample patch is labeled category zero (background) if the percentage of the current patch is zero; otherwise, it is labeled category one (foreground). The fundamental part of any classification operation involves specifying the output or action, as determined based on a given set of inputs or training data. The classification system is formulated as a two-class model: positive patches and negative patches. The positive class contains image patches manually labeled from wheat ears under different illumination intensities. The negative class contains background images manually segmented from soil, rocks, etc. The dataset for the positive training class contains 8647 foreground patches, and that for the negative training class contains 7412 background patches. Meanwhile, the testing set was also generated by the SLIC through the same steps above.

### Multi-feature exaction and combination

(1) **Multi-feature exaction**

Visual features are fundamental for processing digital images to represent image content. A good set of features should contain sufficient discrimination power to discriminate image contents. The feature-extraction section uses color coherence vectors (CCV) as the color feature (Roy and Mukherjee, 2013), the gray level co-occurrence matrix (GLCM) as the texture feature (Varish and Pal, 2018), and introduces the edge histogram descriptor (EHD) feature (Agarwal et al., 2013). Among them, CCV is sufficiently robust to handle background complications and invariants in size, orientation, and partial occlusion of the canopy image. The GLCM feature has good performance in extracting information from local and frequency domains and it can provide good direction selection and scale selection characteristics. The EHD feature can effectively distinguish the images with very high similarity for colors and has good robustness for the color and brightness changes which are the features with very strong stability. Each feature describes the image content from different angles, performing a reasonable optimization and integration to achieve a more comprehensive description of the image content. The final feature matrix contains of three elements: $f_{1}^{\text{C}}$, $f_{2}^{\text{G}}$, and $f_{3}^{\text{E}}$.

**(2) Multi-Feature Combination based on Kernel Principal Component Analysis**

Based on the above description, we obtain a matrix composed of multi-dimensional features. Differences clearly exist for the importance of each feature in the classification process, then reasonably constructing the feature space, so it is important to assign weights to the features according to the importance of features. To achieve this goal, we use KPCA to extract the principal component of features, combining different features to determine the feature weights for the importance of different image classes (Twining and Taylor, 2003). The following details the specific method of classifying feature weights.

We first normalized the fused feature to unify the range of values. The importance of features is inversely proportional to the dispersion of the feature distribution; features with a higher dispersion have a lower importance, which means that a smaller standard deviation leads to a higher importance for features. We thus use *I*_*n*_ to indicate the importance of features:

(1) $$\begin{array}{l} I_{n}=\frac{1}{1+k_{n}} \end{array}$$

Where *k*_*n*_ represents the standard deviation in class *j* of the sample set. When the distribution of one-dimensional features is more concentrated, the standard deviation is smaller, the corresponding *k*_*n*_ is smaller, and the importance of the feature is greater. The formula for calculating the weight of features in each dimension is

(2) $$\begin{array}{l} W_{jn}=\frac{I_{n}}{\overset{N}{\underset{n=1}{\sum }}I_{n}} \end{array}$$

Through the above operation, we can merge the multiple feature vectors into a new feature matrix so that it can be used for machine learning with our own model.

### Image segmentation method based on twin-support-vector-machine segmentation model and noise reduction

We introduce a twin-support-vector-machine segmentation (TWSVM-Seg) model, which is based on the traditional SVM model is better for segmentation of wheat-ear images (Peng et al., 2016). It is similar in form to a traditional SWM with all its advantages. Moreover, it deals better with large-scale data.

In the data *X* ∈ *R*^(m*n)^ to be classified, we take positive samples *m*_1_ with the “1” class from the training set to obtain matrix *A*_*m*_1_·*n*_ We then take negative samples *m*_2_ with the “0” class from the train set to obtain matrix*B*_*m*_2_·*n*_. We obtain a classification plane for each of the two classes. The data that belong to each class are, to the extent possible, near the corresponding classification plane.

The required hyperplane parameters can be obtained by solving the following optimization problem:

(3) $$\begin{array}{l} \begin{array}{c} \underset{w^{\left(1\right)},b^{\left(1\right)}}{\min}\frac{1}{2}||K\left(A,C^{\prime }\right)w^{\left(1\right)}+e_{1}b^{\left(1\right)}|{|}^{2}+c_{1}{e_{2}}^{\prime }q\\ st.-\left(K\left(B,C^{\prime }\right)w^{\left(1\right)}+e_{2}b^{\left(1\right)}\right)+q\geq e_{2},q\geq 0 \end{array} \end{array}$$

(4) $$\begin{array}{l} \begin{array}{c} \underset{w^{\left(2\right)},b^{\left(2\right)}}{\min}\frac{1}{2}||K\left(B,C^{\prime }\right)w^{\left(2\right)}+e_{2}b^{\left(2\right)}|{|}^{2}+c_{2}{e_{1}}^{\prime }q\\ st.\left(K\left(A,C^{\prime }\right)w^{\left(2\right)}+e_{\text{1}}b^{\left(2\right)}\right)+q\geq e_{1},q\geq 0 \end{array} \end{array}$$

where *K* is the kernel function, *A* refers to *m*_1_ positive (wheat ear) samples and *B* refers to *m*_2_ negative (background) samples., e_1_ and e_2_ indicate the unit vector of the corresponding dimension, c_1_ and c_2_ are penalty coefficients, *w* is the normal vector of the optimal hyperplane, and *b* is the offset of the optimal hyperplane. *q* represents the discriminant coefficient. Here, the kernel function *K* is used to populate the TWSVM. Analysis shows that different kernel functions have very large impact on performance of TWSVM, and kernel function is also one of the adjustable parameters in TWSVM. Kernel functions, nuclear parameters and high-dimensional mapping space have a one-to-one relationship, so only select the proper kernel functions, nuclear parameters and high-dimensional mapping space when solving classification problem, we can get the separator with excellent learning and generalization ability. In this paper, we use the radial basis function (RBF) kernel *K* because of its excellent learning ability given large samples and low dimensions. We optimize the parameters of the kernel function after selecting. The error penalty factor *c* and gramma in the RBF are critical factors that impact the performance of the TWSVM, so these parameters strongly influence the classification accuracy and generalization ability of TWSVM. Here, we use the grid-search method to optimize and select parameters to obtain the global optimum results. Thus, the linear non-separable problem can be solved. Each sample in the training set belongs only to one of the two classes.

By solving Equations (3) and (4), we get the following two hyperplanes:

(5) $$\begin{array}{l} K\left(x^{\prime },C^{\prime }\right)w^{\left(1\right)}+b^{\left(1\right)}=0 \end{array}$$

(6) $$\begin{array}{l} K\left(x^{\prime },C^{\prime }\right)w^{\left(2\right)}+b^{\left(2\right)}=0 \end{array}$$

The two hyperplanes correspond to two different classes. For a sample to be classified, the distance to these two hyperplanes must be calculated. For each sample, the distance to each hyperplane is compared and the sample is classified into the nearest class.

Through the above operation, the pixels in the test samples could be divided into two classes (wheat ear = 1, background = 0), which generates a binary image to achieve image segment.

After all these operations, we then use the median filter *w* to minimize the noise and remove the result of burrs and noise over the binary image (Igoe et al., 2018). For this, we slide a window size of three pixels over the entire image, pixel by pixel, and numerically sort the pixel values in the window and replace them with a median value of neighboring pixels.

This process provides several separate and disconnected bright areas, each of which represents an unidentified wheat ear. Here, we use the regionprops function in Matlab R2017b (Mathworks Inc., Massachusetts, USA) to count the independent regions in the image, which corresponds to counting the number of wheat ears. In addition, we apply the ground truth function to each image, and manually label the wheat ears in the image so as to compare with the result of computer recognition.

### Criteria to evaluation algorithm

To evaluate the quality of the segmentation, we use the six indicators *Qseg, Sr*, structural similarity index (SSIM), Precision, Recall, and the F-measure. The following is a detailed description of the meaning and range of each index (Xiong et al., 2017).

*Qseg*, which is based on both plants and background regions, ranges from 0 to 1. The closer *Qseg* is to unity, the more accurate is the segmentation. Thus, *Qseg* reflects the consistency of all the image pixels, including foreground ear part and the background part. *Sr* represents the consistency of only the ear part of the image. From the perspective of an image, it reflects the completeness of the segmentation results. The SSIM describes the degree of similarity between the segmentation images and the ground truth images. The SSIM ranges from 0 to 1, with higher values indicating more similarity between images. Precision and Recall are the most basic indicators for revealing the final segmentation results. Precision illustrates the accuracy of the segmentation algorithm, and Recall represents the completeness of the segmented images. In practice, Precision and Recall interact with each other. When Precision is high, Recall is low. The F-measure is proposed to balance these two indicators. The higher the value of the F-measure, the better the segmentation results. Table 1 shows how to calculate these indicators.

**Table 1:** Formulas to calculate criteria for evaluating segmentation precision.

**Evaluation criteria**	**Calculation formula**
*Q_*seg*_*	(7) $Q_{seg}=\frac{\overset{a}{\underset{i=0}{\sum }}\overset{b}{\underset{j=0}{\sum }}\left(M{\left(\omega \right)}_{i,j}\cap N{\left(\omega \right)}_{i,j}\right)}{\overset{a}{\underset{i=0}{\sum }}\overset{b}{\underset{j=0}{\sum }}\left(M{\left(\omega \right)}_{i,j}\cup N{\left(\omega \right)}_{i,j}\right)}$
*S_*r*_*	(8) $S_{r}=\frac{\overset{a}{\underset{i=0}{\sum }}\overset{b}{\underset{j=0}{\sum }}\left(M{\left(\omega \right)}_{i,j}\cap N{\left(\omega \right)}_{i,j}\right)}{\overset{a}{\underset{i=0}{\sum }}\overset{b}{\underset{j=0}{\sum }}N{\left(\omega \right)}_{i,j}}$
Precision	(9) $Precision=\frac{TP}{TP+FP}$
Recall	(10) $Recall=\frac{TP}{TP+FN}$
F-measure	(11) $F=\frac{2\times Precision\times Recall}{Precision+Recall}\%$

In Equations (7) and (8), *M* represent the ear pixels (with ω = 255) or background pixels (with ω = 0). *N* in Equations (1) and (2) represents a reference set of manually segmented ear pixels (with ω = 255) or background pixels (with ω = 0). *a* and *b* give the row and column of the image and *i, j* give the pixel coordinate of the image. In Equations (9–11), *TP, TN, FP*, and *FN* are the number of true positives, true negatives, false positives, and false negatives, respectively. True positives (*TP*) means when the predicted results and the corresponding ground truth are both wheat ear pixels. True negatives (*TN*) are when the predicted results and the corresponding ground truth are both background pixels. False positives (*FP*) are the pixels that are classified as wheat ear pixels, but the ground truth of those pixels is background. False negatives (*FN*) are the pixels that belong to the ground truth but are not correctly discriminated.

## Results

The performance of the proposed machine learning method is evaluated based on comparing its results against manual measurements. The algorithms were developed in Matlab R2017b. Segmenting a 3500 × 1800 image takes only 0.1 s on average on a Windows 10 PC with 4-core Intel Core i5 processor (2.71 GHz) with 12 GB RAM. For this paper, we separated the image dataset of 300 plots into three categories of equal size with different illumination conditions and show their segmentation results and corresponding ground truths.

### Results of several image-segmentation methods

We apply three traditional segmentation methods to compare their results with those of the proposed method. The unsupervised methods are the Otsu method, mean shift and normalized cuts (MSNC), and the edge-region active contour model.

**Figure 4:**
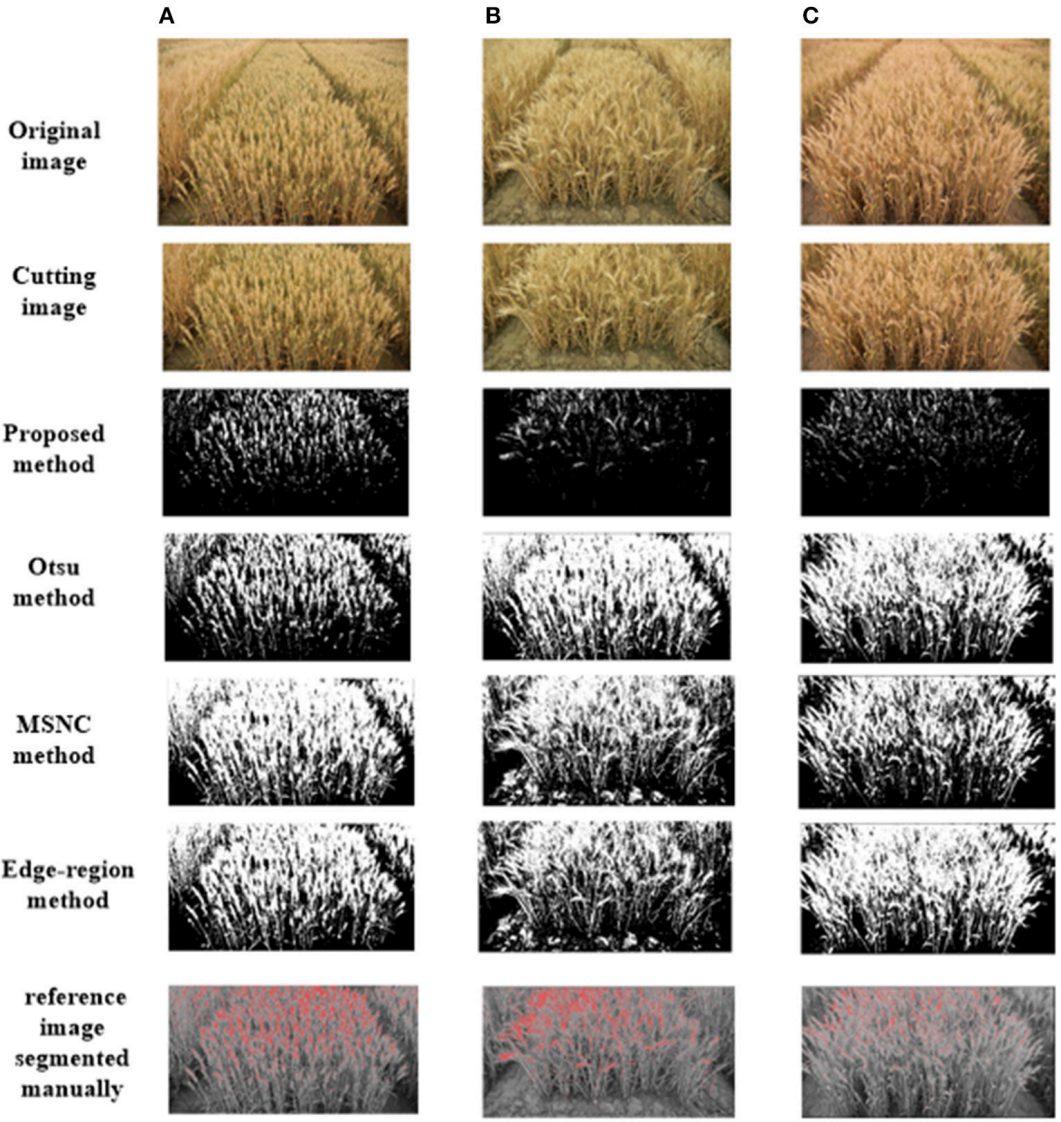
Examples of test images after segmentation and their corresponding ground truths. The test images are randomly selected from the image dataset under different illumination conditions: **(A)** high illumination intensity, **(B)** medium illumination intensity, **(C)** low illumination intensity.

The Otsu method is a global thresholding method. The Otsu threshold is found by searching across the entire range of pixel values of an image until the intra-class variances are minimized. MSNC first applies the mean shift algorithm to obtain subgraphs and then applies the normalized cut. Currency denomination and detection is an application of image segmentation. The edge-region active contour model consists of two main energy terms: an edge-region term and a regularization term. This model not only has the desirable property of processing inhomogeneous regions but also provides satisfactory convergence speed (Cheng et al., 2001) (Figure 4).

Figure 4 shows the input and output images that there are mainly three cases where these method has not worked properly: (i) pixels labeled as ear actually corresponded to leaves; (ii) contrast between the ear and soil was not great enough and (iii) whereas the algorithm labeled the area as an ear, those pixels are noise.

**Figure 5:**
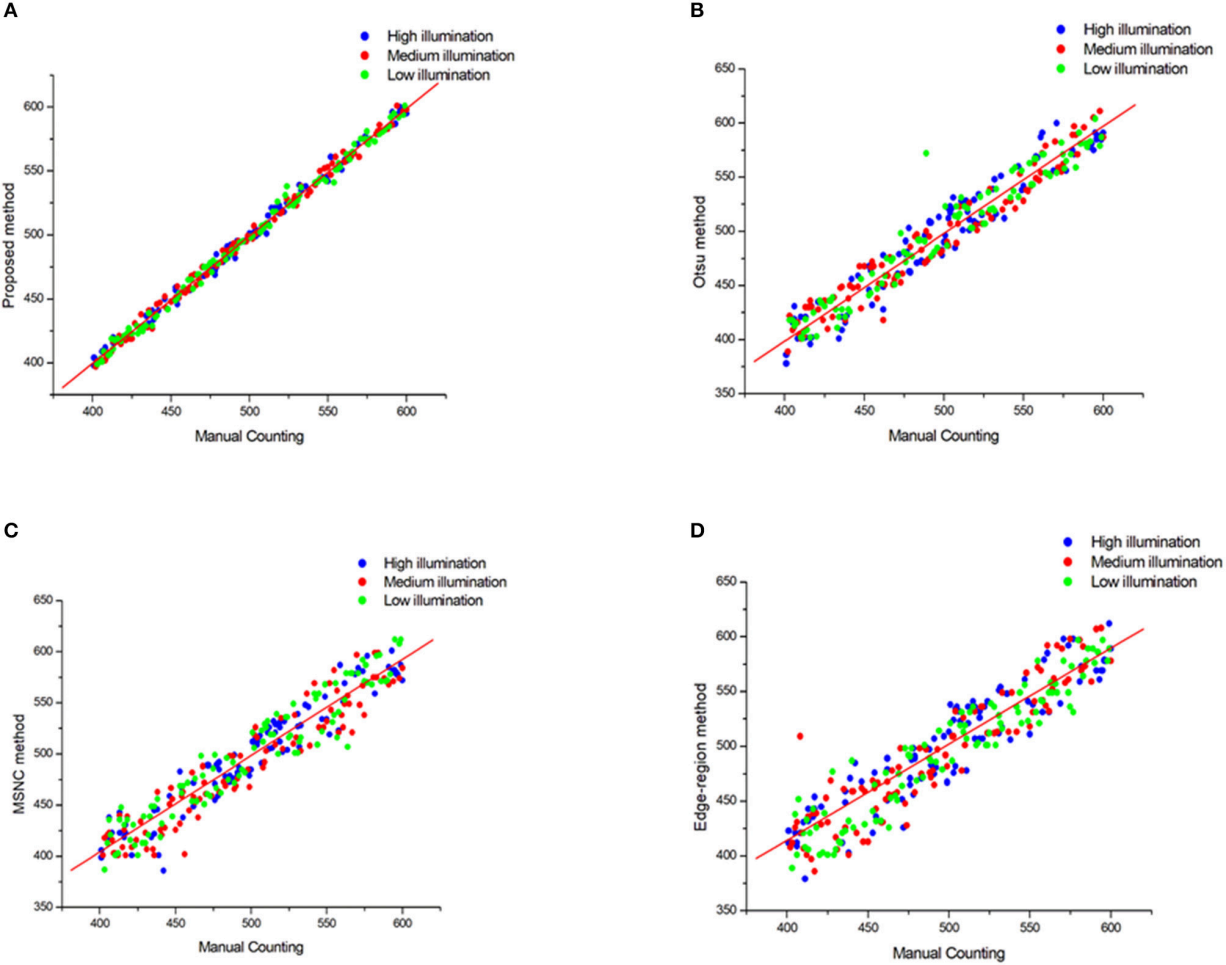
Plots of Mannual counting with different segmentation method in different illumination. **(A)** Proposed method, **(B)** Otsu method, **(C)** MSNC method and **(D)** Edge-region method.

Furthermore, the linear regression between the manual counting and the algorithm counting was calculated for 300 plots with different illumination (Figure 5, Table 2).

**Table 2:** Results of counting wheat ears by using different segmentation methods in different illumination conditions.

	**Proposed method**	**Otsu method**	**MSNC method**	**Edge-region method**
High	R^2^ = 0.99	R^2^ = 0.92	R^2^ = 0.90	R^2^ = 0.92
illumination	SD = 4.07	SD = 15.82	SD = 15.72	SD = 15.82
Medium	R^2^ = 0.99	R^2^ = 0.94	R^2^ = 0.90	R^2^ = 0.85
illumination	SD = 4.07	SD = 13.33	SD = 18.10	SD = 22.05
Low	R^2^ = 0.99	R^2^ = 0.94	R^2^ = 0.90	R^2^ = 0.88
illumination	SD = 4.08	SD = 14.22	SD = 17.29	SD = 18.35
Total result	R^2^ = 0.99	R^2^ = 0.94	R^2^ = 0.90	R^2^ = 0.86
	SD = 4.05	SD = 14.44	SD = 17.21	SD = 20.57

We see from Figure 5 and Table 2 that the results of the proposed method correlate strongly (***R***^2^ = 0.99) with the manual measurements for all selected images. Moreover, the standard deviation (***SD***) between the test set is smallest which means that the proposed method is the most stable. But the simple use of the correlation index cannot accurately evaluate the recognition accuracy, so we introduce below more evaluation criteria to verify the performance of these methods.

We can draw a conclusion from the Figure 4 that there are mainly three kinds of regions in the image indicating examples where the algorithm has not worked properly: (a) Region 1 shows the case where two ears overlap together and are considered as one; (b) In Region 2, false negatives resulted in wheat ears that were not detected by the algorithm because the contrast between the wheat ear and soil was not great enough and the segmentation algorithm discarded that region; (c) In Region 3, whereas the algorithm labeled the area as a wheat ear, those targets are noise being a result of background brightness caused by a foreign object.

**Figure 6:**
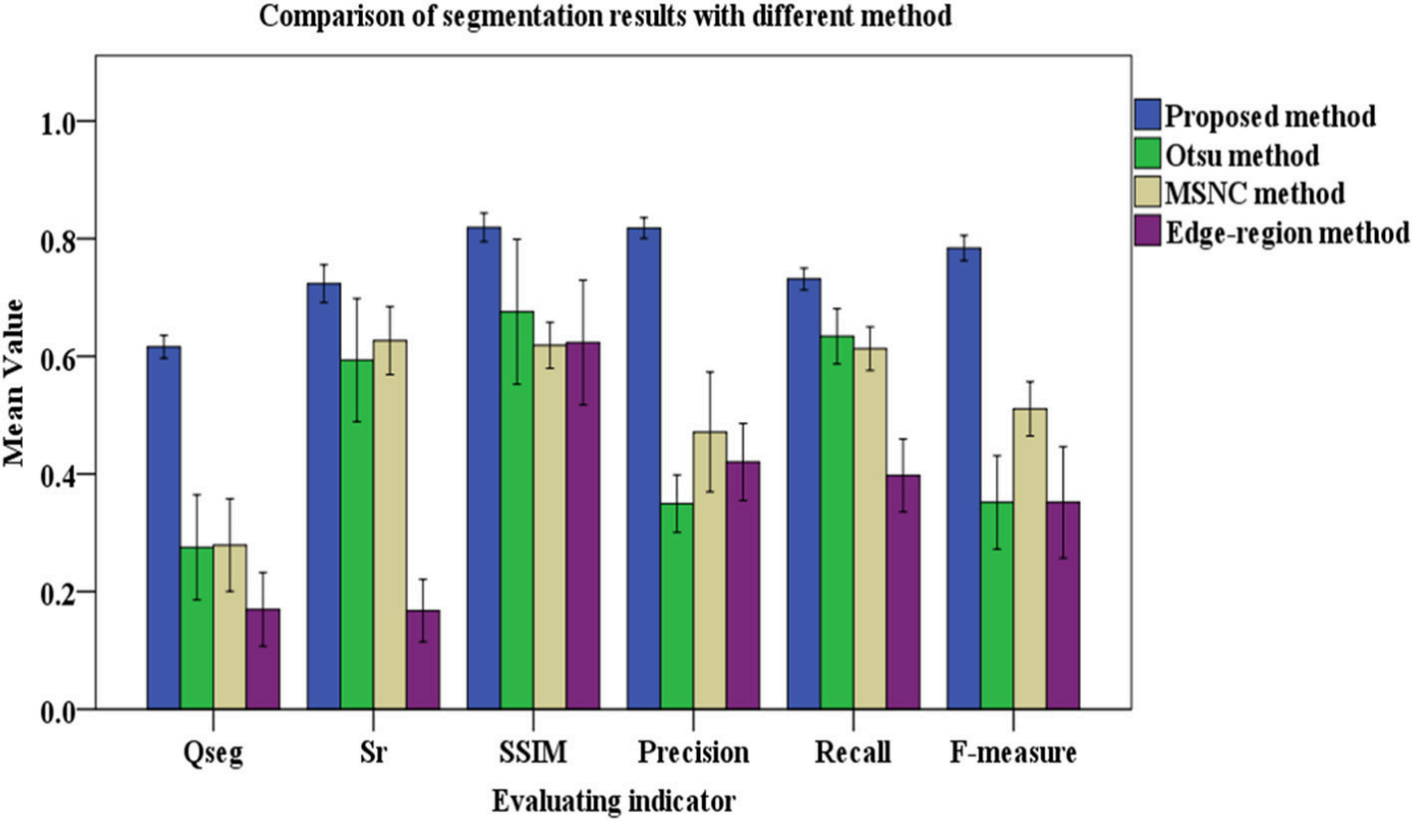
Results of evaluating segmentation with different methods. The color columns represent the means value and the black lines represent the standard deviations for the test images. In addition, the color differences between columns refers to the categories of segmentation methods. Blue is for the proposed method, green is for the Otsu method, yellow is for the MSNC, and purple is for the edge-region active contour model.

Comparing the manual counting results with the statistical results obtained by different segmentation methods gives satisfactory results. To evaluate the segmentation results more comprehensively, six indices were introduced to judge the effect of the segmentation (Figure 6).

Figure 6 shows that, compared with other three common methods mentioned in this paper, the proposed method gives the maximum mean value of the six indicators. The mean values of *Qseg, Sr*, SSIM, Precision, Recall and F-measure (%) are 0.62, 0.72, 0.82, 0.82, 0.73, and 0.73, respectively. Moreover, Figure 6 shows that the proposed method gives the minimum standard deviation for each evaluation index, which means that it gives the most stable performance with images under different illumination conditions.

### Results of segmentation accuracy with different classifiers

**Figure 7:**
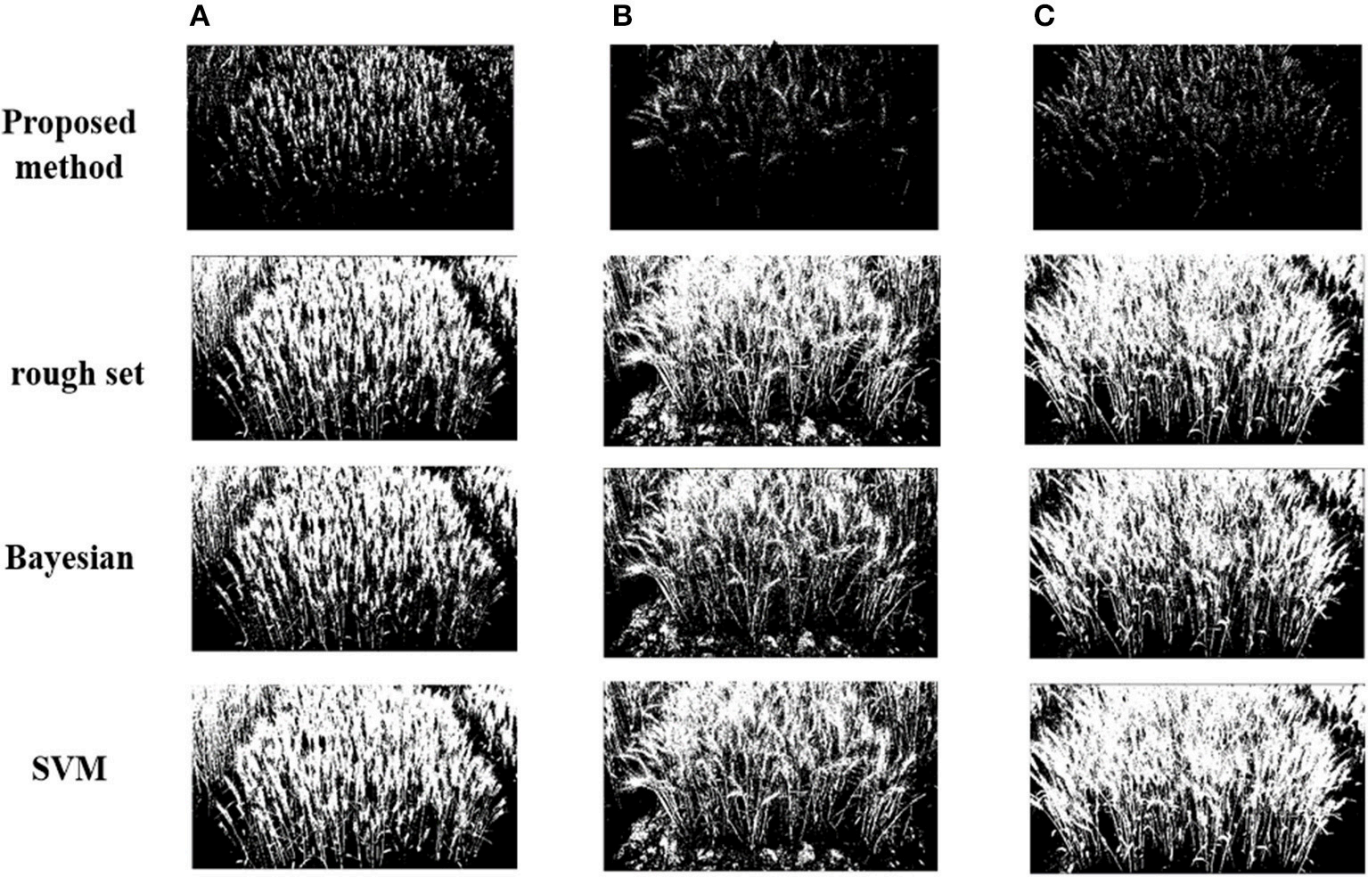
Segmentation results for different classifiers under different illumination conditions: **(A)** high illumination intensity, **(B)** medium illumination intensity, **(C)** low illumination intensity.

**Figure 8:**
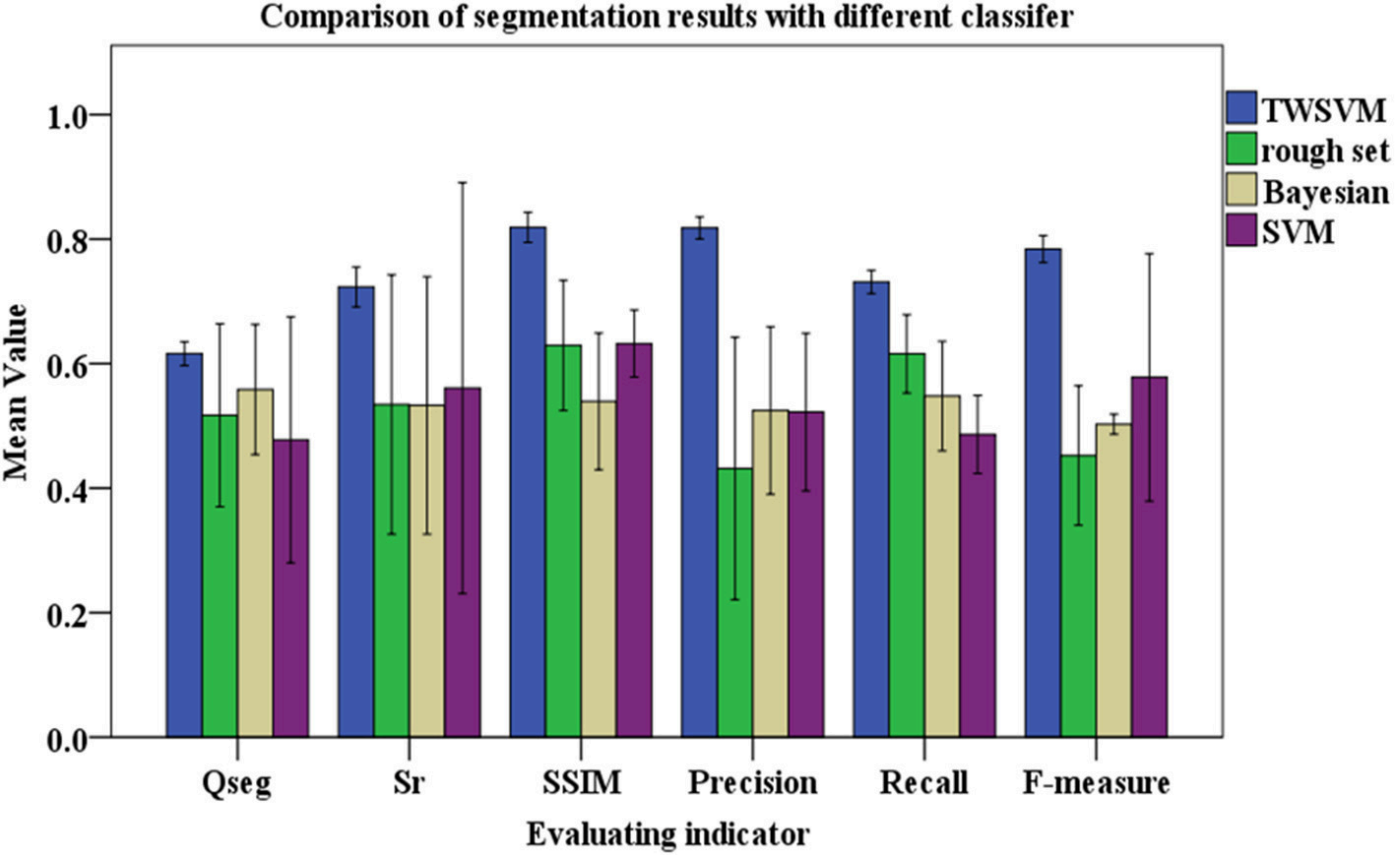
Comparison of segmentation with different classifiers. The color columns represent the mean values and the black lines represent the standard deviations for the testing images. Blue represent TWSWM, green is for rough set, yellow is for Bayesian, and purple is for SVM.

Differences in selecting the classifier can lead to quite different segmentation precision. To verify the proposed algorithm (TWSVM), we compare it against three well-established algorithms: rough set, Bayesian, SVM (Figures 7, 8).

Figure 8 shows that the TWSVM provides better segmentation, and the wheat-ear integrity is well maintained. Except for the TWSVM, the SD of the other three algorithms is relatively large, which reflects their weak adaptability to different field testing images. In addition, the average of *Q*_*seg*_ for the proposed algorithm is about 0.626, which is significantly greater than for the other three algorithms. Thus, the proposed algorithm is more consistent for both the panicle foreground part and the background part. In addition, the mean value of the SSIM for the proposed algorithm is greater than that of the other three contrast algorithms. Moreover, the F-measure is a comprehensive indicator and accounts for Precision and Recall; it is as high as 0.738 using our proposed algorithm compared with 0.398, 0.452, and 0.578 for the other algorithms, respectively. These results show that the proposed algorithm accurately segments the wheat ears and guarantees the integrity of segmentation.

### Results of recognition accuracy with different image features

**Figure 9:**
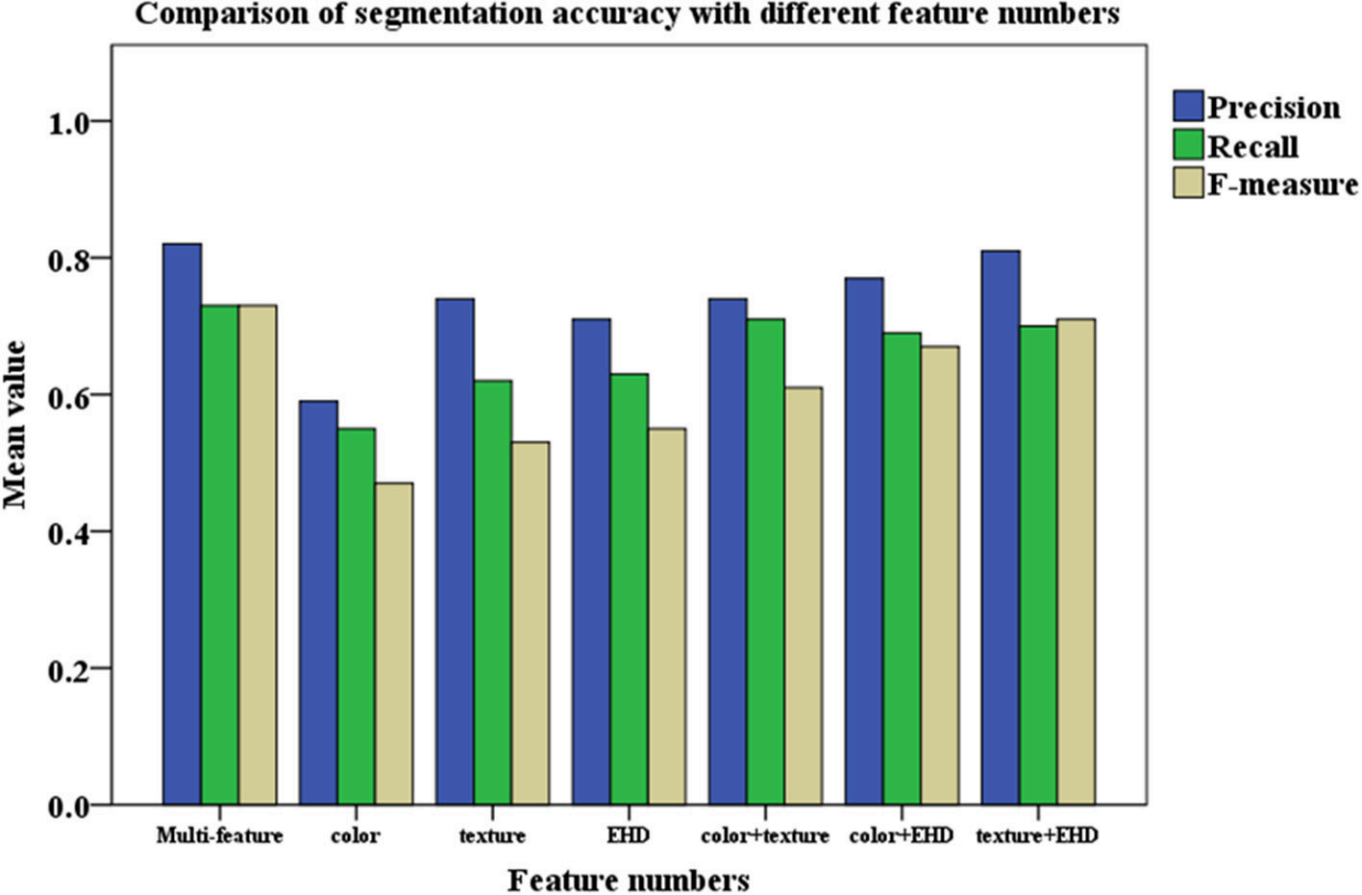
Comparison of segmentation accuracy with different feature numbers. The color columns represent the means value. The color differences between columns means the categories of evaluating indicator. [Precision (Blue), Recall (Green), and F-measure (Yellow)].

The color feature, texture feature, and EHD feature are optimized to perform the segmentation of images, in Figure 8, the segmentation testing results by using different number of features are given (Figure 9).

Results are found by Figure 9 that for each class of wheat ear image, the segmentation accuracy of the proposed algorithm is obviously better than that when using a single feature. And it can be seen that for the selected color feature, texture feature, and EHD feature, each feature has very different segmentation results, which also shows that there is a complementary relationship between each feature. After optimizing each feature, the proposed algorithm gives the weight to each feature, each sample constructs a reasonable feature space, and the average precision of the whole image is more than 82%, which is 12.4, 7.5, and 9.2% higher than the recognition accuracy of color feature, texture feature, and EHD feature, respectively.

We use *Q*_*seg*_, and *S*_*r*_ to judge the segmentation accuracy; the results are given in Table 3.

**Table 3:** Comparison of mean accuracy rate between multi-feature and single feature.

	**Multi-feature**	**Color**	**Texture**	**EHD**	**Color+Texture**	**Color+EHD**	**Texture+EHD**
*Q_*seg*_*	0.62	0.41	0.52	0.47	0.54	0.55	0.58
*S_*r*_*	0.72	0.49	0.55	0.67	0.71	0.70	0.68

As shown in Table 3, the use of multiple features is more robust against background noise and variations in illumination. As a result, we select the multi-feature method as the optimum technique and compare it with the state-of-the-art vegetation segmentation described herein.

Moreover, robust hue histograms (**RHH**) (color) and Scale-invariant feature transform (**SIFT**) (texture) were used as two typical features to participate in comparative experiments to verify the reliability of feature selection (van de Sande et al., 2010; Seeland et al., 2017). Here, CCV and GLCM were replaced with RHH and SIFT in order to test the variation of precision after different combination of features (Table 4).

**Table 4:** Comparison of mean accuracy rate between different feature combination strategy.

	**Proposed**	**RHH**	**SIFT**
*Q_*seg*_*	0.62	0.60	0.63
*S_*r*_*	0.72	0.73	0.71
SSIM	0.82	0.72	0.69
Precision	0.82	0.78	0.71
Recall	0.73	0.65	0.61
F-measure	0.73	0.68	0.67

Table 4 could provide a conclusion that the combination of features given in this paper could get better segmentation effect. Although on some indices, for example, *Q*_*seg*_ and *S*_*r*_, the proposed feature combination strategy was slightly lower than the match group (< 5%). In general, it usually gives more accurate results especially in Precision and F-measure.

## Discussion

To be relevant for high-throughput phenotyping in field conditions, the segmentation algorithms must be sufficiently robust to handle dynamic illumination conditions and complex canopy architecture throughout the entire observation period. We find that the recognition accuracy of the classifiers differs substantially depending on the number of features and the illumination intensity. Here, we analyze how these factors affect the accuracy of the segmentation results, respectively.

### Effect of illumination intensity and shadow on recognition accuracy

**Figure 10:**
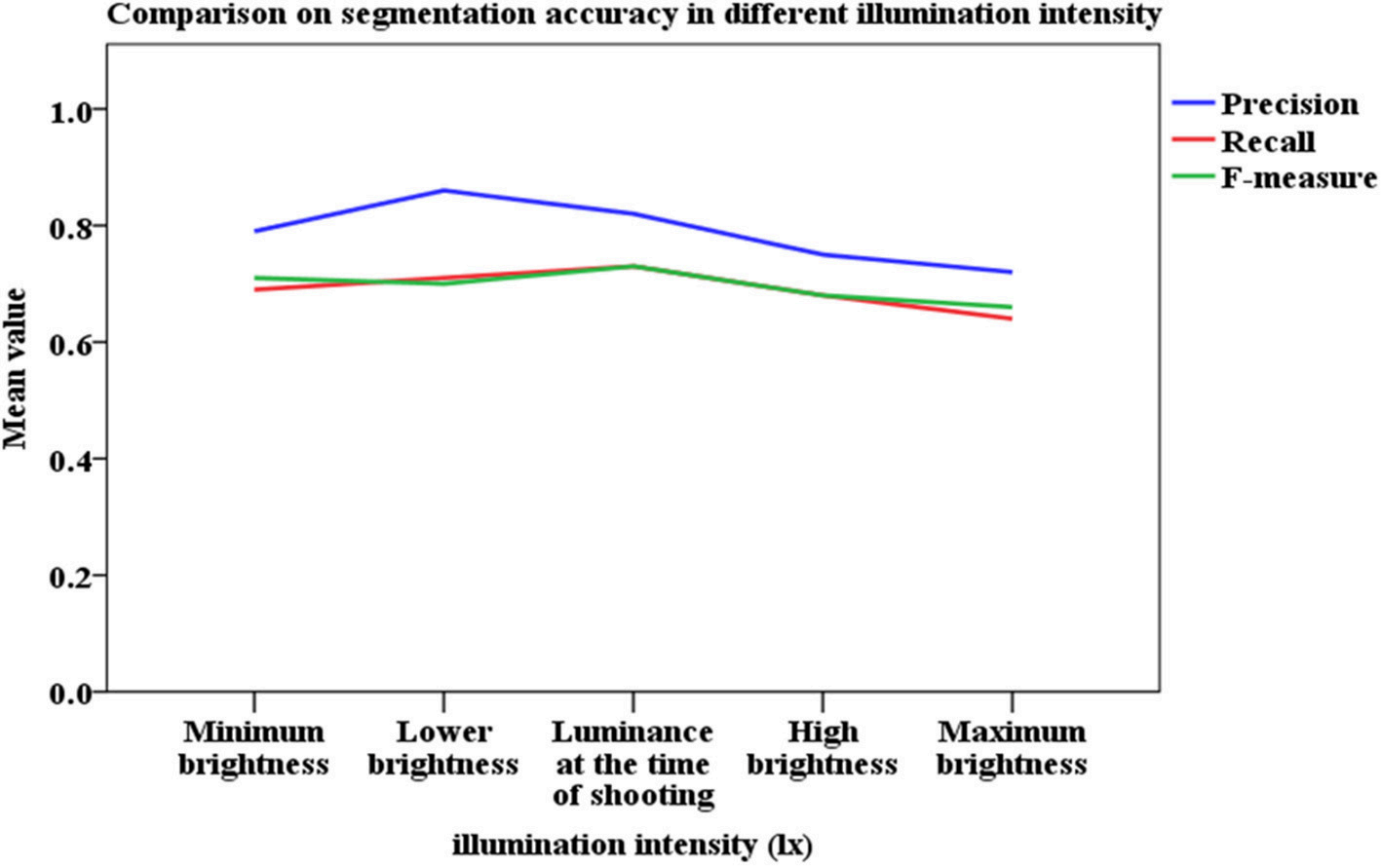
Comparison of segmentation accuracy under different illumination intensities.

Analyzing images acquired outdoors is a challenging task because the ambient illumination varies throughout the growing season. Unlike single plants grown in pots in greenhouse facilities, segmenting the vegetation from a field-grown plot is complex because of overlapping leaves and because portions of the canopy are shadowed or have high specular reflectance, each of which contribute to underestimating vegetation pixels in an image. To study the robustness of the method under different illumination conditions, we use the image brightness adjustment function of Photoshop CS6 (Adobe Systems Incorporated, California, USA) to adjust the luminance components. The original image brightness is called the “central value of brightness adjustment,” and the image results of five different luminance conditions are simulated by varying from dark to bright. The results are then associated with the artificial recognition results by using the proposed method to determine how the different illumination conditions affect this recognition method (Figure 10).

Figure 10 shows that the segmentation accuracy reaches the highest value under conditions of lower brightness, which corresponds to overcast sky without overexposure or underexposure. We thus conclude that the illumination condition affects the recognition accuracy.

Unlike the use of artificial light and enclosures, our flexible and fast image acquisition technique presents some major challenges related to image processing. Sharp shadows and bright surfaces may appear in the images as a product of the light conditions. As such, in order to provide robust results, the image processing algorithm pipeline must consider effects related with shadows. When the training set is set up, the images under different shading conditions are included. The recognition results in Figure 5 and Table 2 show that there is not much difference in the recognition results under different shading conditions.

### Analysis of effect of noise on recognition accuracy

**Figure 11:**
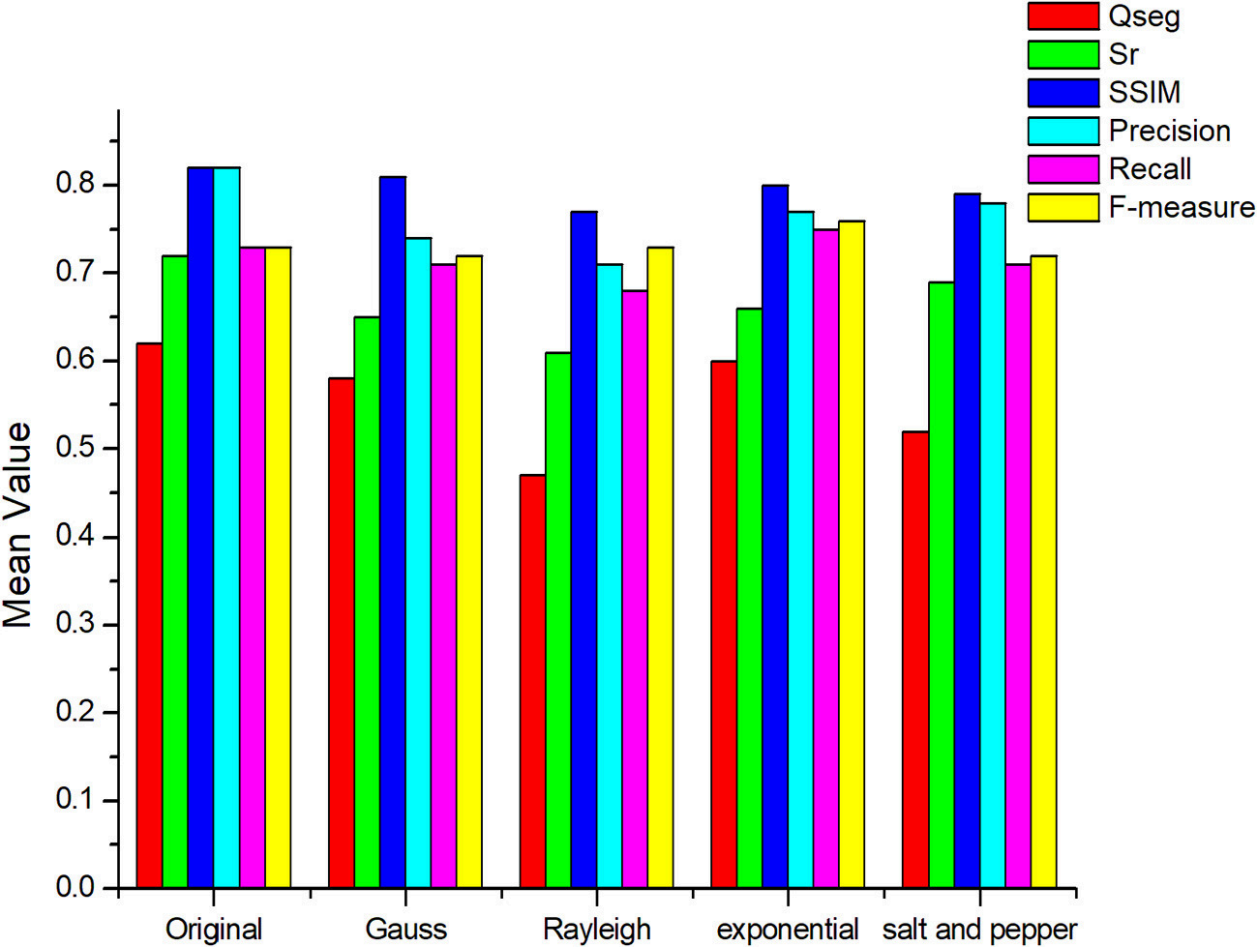
Analysis of the noise resistance of the proposed method.

Noise may be generated through the entire process of the image processing and may be divided into two categories: system noise and environmental noise. System noise is usually caused by the imaging system itself and includes electronic noise and photoelectron noise. Environmental noise is caused by a poor image-acquisition environment and unreasonable image-acquisition methods. The proposed method relies on counting disconnected regions and fitting the obtained number to the manually counted amount of wheat ears via linear regression. So the excessive noise points will increase the error in statistical results. Here, Gauss noise, Rayleigh noise, exponential noise, and salt-and-pepper noise were introduced to test the noise robustness of the proposed method (Figure 11).

Figure 11 shows that noise affects the accuracy of segmentation. Specifically, Rayleigh noise and salt-and-pepper noise reduce the accuracy by over 40% whereas the two other types of noise have little effect on the result (< 20%). The first two types of noise are denser and larger and are easily mistaken for wheat ears. However, a *Median filter* or a *Laplacian filter* can effectively filter out these two types of noise and may be considered for denoising in actual production.

### Effect of different camera angles and field-of-view on recognition accuracy

**Figure 12:**
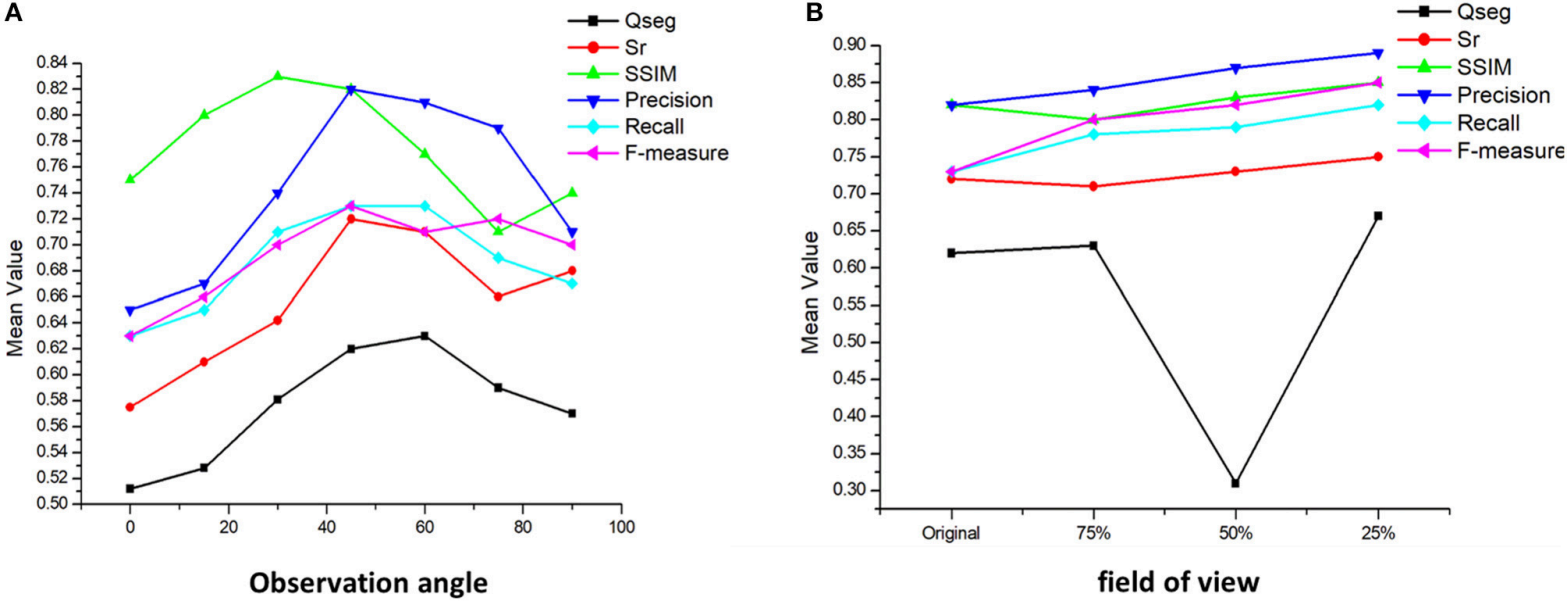
Analysis of different camera angles and field-of-view on recognition accuracy. **(A)** The variation of accuracy under different observation angles; **(B)** The variation of accuracy under different field of view.

The performance of the algorithm was further tested through the different camera angles and fields of view. First, the images are taken at six different angles: 90, 75, 60, 45, 30, 15, and 0° under same light intensity and camera parameters. Then, the center of each image is taken as the center of shooting, and the image is cut at 1/2, 1/4, and 1/8 long sides, respectively, then the imaging results of different fields of view are obtained. We use the same algorithm pipeline proposed for different camera angles and field-of-view images. As before, manual image-based counting is used as the validation data (Figure 12).

The different camera angles show, with respect to the original images taken at 45°, a decrease of over 20% in success rate while the shooting angle is close to 0°. The interference of leaves and stems and the mutual occlusion between ears make it impossible to get an accurate number near the horizontal position. Meanwhile, the accuracy of image recognition from the vertical angle is also reduced by about 15%. The significant difference in wheat morphology between vertical and oblique observations may at the origin of this result.

The different field-of-view results show an increase of 8% in success rate when the images are reduced to 75% of their original size. Success rates increased by a maximum of 13 and 15% for image size divided by 50 and 25% values, respectively. Near the edge of the image, distortion of the wheat ear shape reduces the recognition accuracy. At the same time, other interference factors affect the edge parts, such as noise, which will also affect the final result. In future work, the proper range of field of view should be studied.

### Analysis of algorithm efficiency

**Figure 13:**
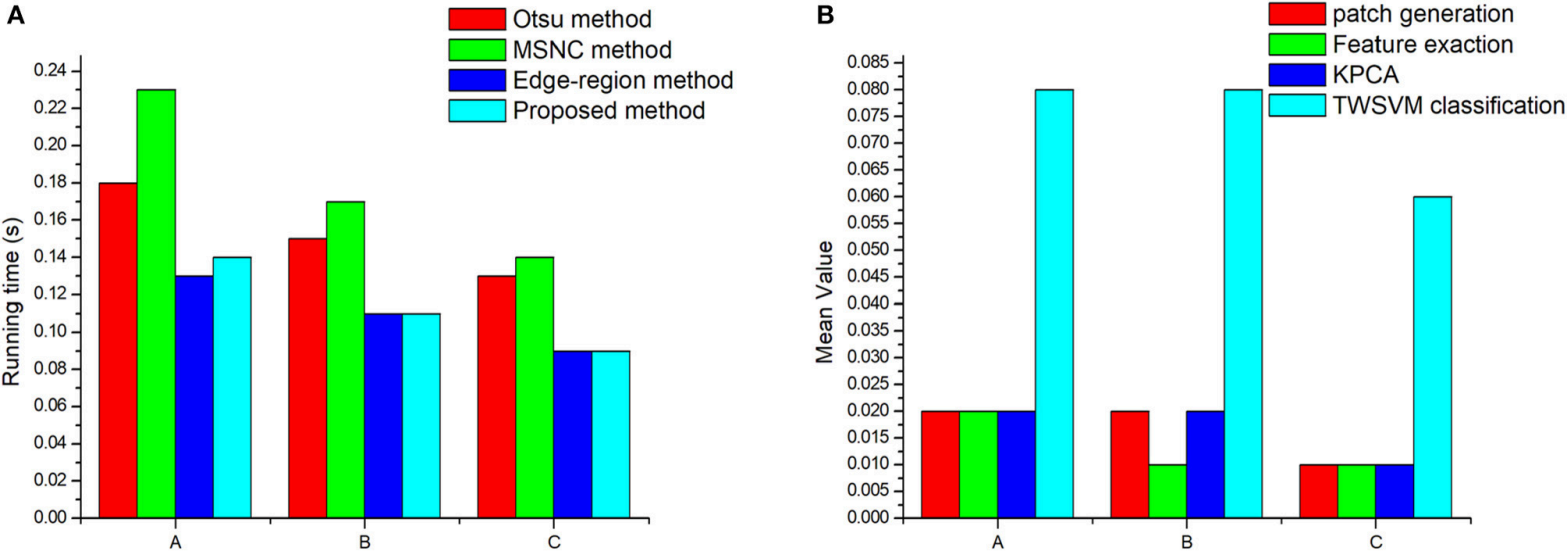
Algorithm efficiency analysis: **(A)** Operating efficiency of different segmentation methods. **(B)** The running time of each step of the proposed method. “A” represents the images obtained under low intensity illumination, “B” represent images obtained under normal illumination conditions, and “C” represent images obtained under high-intensity illumination.

We use the average running time of each segmentation method as a metric for the efficiency of the algorithm (Figure 13).

We conclude from Figure 13 that the average running time of the proposed algorithm is 0.1 s for calculating the number of wheat ears in a single scene, which means that the proposed algorithm is an efficient method. Moreover, the running time increases as the number of wheat ears increases (compare Table 3 and Figure 13A). It seems that the increase in the number of target objects may lead to an increase in the time complexity of the algorithm. Thus, the proposed method may be used as a high-throughput post processing method to measure seeding statistics for large-scale breeding programs. We can also draw a conclusion from the Figure 13B that the most time-consuming step is patch classification by TWSVM. An intuitive improvement to further improve algorithm efficiency would be to parallelize all the procedures. However, such an improvement is likely to be hardware limited (due to the input-output speed of the memory and hard drive).

To summarize, the proposed machine learning approach offers the advantage of versatility and can extract the number of green vegetation, such as wheat, maize, etc. Given an adequate training dataset, it could even detect disease or pest symptoms. As already mentioned, the performance of any supervised learning model strongly depends on the training datasets. Therefore, to have a good model, a substantial set of training data is important. Acquiring a training data is time consuming and can be subjective. Our aim is to expand this study by integrating a semi-adaptive approach to semi-automatically generate larger and more reliable training datasets. In addition, we must test the model on more varieties and different crops.

## Conclusion

Accurately estimating wheat yield requires accurate statistics of the number of wheat ears per unit area. This is achieved in this study by using a method for automatic segmentation of target plant material in RGB images of wheat ears and by splitting these images into individual targets. The initial step in this proposed method requires minimal manual intervention to generate patches from the original images. This technique is partially verified by comparing its results with those of manual and automated measures of image segmentation. The good correlation between manual and automated measurements confirms the value of the proposed segmentation method. The segmentation performance is evaluated in this way because manual image segmentation is labor intensive and subject to observer bias. Manual inspection of segmented images indicates good quality segmentation in all images. Compared with other approaches, the proposed algorithm provides better segmentation and recognition accuracy. Moreover, this method can be expanded for use in different field environments and with different light intensities and soil reflectance.

## Author contributions

CZ and GY anlayzed the data and drafted the article. DL directed images processing. XY and HY designed the experiments, and JY conducted the field measurements. All authors gave final approval for publication.

### Conflict of interest statement

The authors declare that the research was conducted in the absence of any commercial or financial relationships that could be construed as a potential conflict of interest.
